# Thymoglobulin, interferon-γ and interleukin-2 efficiently expand cytokine-induced killer (CIK) cells in clinical-grade cultures

**DOI:** 10.1186/1479-5876-8-129

**Published:** 2010-12-07

**Authors:** Giuseppina Bonanno, Paola Iudicone, Andrea Mariotti, Annabella Procoli, Annino Pandolfi, Daniela Fioravanti, Maria Corallo, Alessandro Perillo, Giovanni Scambia, Luca Pierelli, Sergio Rutella

**Affiliations:** 1 Department of Gynecology, Catholic University Med. School, Rome, Italy; 2 Department of Blood Transfusion and Cell Therapy, Azienda Ospedaliera "S. Camillo-Forlanini", Rome, Italy; 3 Department of Experimental Medicine, University Sapienza, Rome, Italy; 4 Department of Hematology, Catholic University Med. School, Rome, Italy; 5 IRCCS San Raffaele Pisana, Rome, Italy

## Abstract

**Background:**

Cytokine-induced killer (CIK) cells are typically differentiated *in vitro *with interferon (IFN)-γ and αCD3 monoclonal antibodies (mAb), followed by the repeated provision of interleukin (IL)-2. It is presently unknown whether thymoglobulin (TG), a preparation of polyclonal rabbit γ immunoglobulins directed against human thymocytes, can improve the generation efficiency of CIK cells compared with αCD3 mAb in a clinical-grade culture protocol.

**Methods:**

Peripheral blood mononuclear cells (PBMC) from 10 healthy donors and 4 patients with solid cancer were primed with IFN-γ on day 0 and low (50 ng/ml), intermediate (250 ng/ml) and high (500 ng/ml) concentrations of either αCD3 mAb or TG on day 1, and were fed with IL-2 every 3 days for 21 days. Aliquots of cells were harvested weekly to monitor the expression of representative members of the killer-like immunoglobulin receptor (KIR), NK inhibitory receptor, NK activating receptor and NK triggering receptor families. We also quantified the frequency of *bona fide *regulatory T cells (Treg), a T-cell subset implicated in the down-regulation of anti-tumor immunity, and tested the *in vitro *cytotoxic activity of CIK cells against NK-sensitive, chronic myeloid leukaemia K562 cells.

**Results:**

CIK cells expanded more vigorously in cultures supplemented with intermediate and high concentrations of TG compared with 50 ng/ml αCD3 mAb. TG-driven CIK cells expressed a constellation of NK activating/inhibitory receptors, such as CD158a and CD158b, NKp46, NKG2D and NKG2A/CD94, released high quantities of IL-12p40 and efficiently lysed K562 target cells. Of interest, the frequency of Treg cells was lower at any time-point compared with PBMC cultures nurtured with αCD3 mAb. Cancer patient-derived CIK cells were also expanded after priming with TG, but they expressed lower levels of the NKp46 triggering receptor and NKG2D activating receptor, thus manifesting a reduced ability to lyse K562 cells.

**Conclusions:**

TG fosters the generation of functional CIK cells with no concomitant expansion of tumor-suppressive Treg cells. The culture conditions described herein should be applicable to cancer-bearing individuals, although the differentiation of fully functional CIK cells may be hindered in patients with advanced malignancies.

## Introduction

Adoptive cellular immunotherapy aims at restoring tumour-cell recognition by the immune system, leading to effective tumour cell killing. A major hurdle to the successful immunotherapy of cancer is represented by the difficulty in generating clinically relevant numbers of immune effector cells with potent *in vivo *anti-tumour activity, especially in heavily pre-treated patients. To date, various populations of cytotoxic effector cells have been expanded using robust cell culture procedures and have been administered in a variety of human cancers. Host effector cells endowed with killing activity against tumour cells were initially described in the early 1980s as lymphokine-activated killer (LAK) cells [1,2]. The LAK cell population is heterogeneous, being comprised of CD3^-^CD56^+ ^NK cells, CD3^+^CD56^+ ^MHC-unrestricted cytotoxic T cells and CD3^+^CD56^- ^T cells. Over the years, improvements in culture conditions, such as the addition of αCD3 (OKT3) monoclonal antibody (mAb) at the initiation of culture and the provision of cytokines at the end of culture, translated into better expansion of LAK cells. Current protocols to differentiate cytokine-induced killer (CIK) cells are based on a combination of 1,000 IU/ml interferon (IFN)-γ on day 1 of culture, followed 24 hours later by OKT3 at 50 ng/ml and interleukin (IL)-2 at 300 IU/ml [3]. At the end of the 21-28 day culture period, CD3^+^CD56^+ ^cells, derived from CD3^+^CD56^- ^cells, acquire cytotoxicity against various tumour cell targets, including acute myeloid leukaemia (AML), chronic myeloid leukaemia (CML), B and T-cell lymphoma. The expression of CD56 on CIK cells is thought to result from IFN-γ priming with IL-12 production from monocytes. CIK cells share phenotypic and functional properties of both T cells and NK cells, insofar they express CD3 and are rapidly expandable in culture like T cells, while not necessitating functional priming for *in vivo *activity like NK cells. Interestingly, CIK cells do not recognize target cells through the T-cell receptor (TCR) and do not require the presence of major histocompatibility complex (MHC) molecules on target cells, as suggested by the observation that cytotoxicity is not affected by antibody masking of the TCR or MHC class I or class II molecules [4]. Cytotoxicity by CIK cells does not rely on antibody-dependent cell cytotoxicity (ADCC) mechanisms, given the absence of CD16 on their surface membrane, and is not inhibited by the immune suppressive drugs cyclosporine A and FK506 [5]. Conversely, the anti-tumour activity of CIK cells mainly relies on the engagement of NK Group 2, member D (NKG2D) by NKG2D ligands on tumour cells, and on perforin-mediated pathways [6].

The *in vivo *activity of CIK cells was initially demonstrated in a murine SCID/human lymphoma model, where the co-administration of CIK cells with B lymphoma cells exerted a favorable effect on mice survival, with a 1.5-2-log cell kill and minimal toxicity against normal hematopoietic precursors [4]. CIK cells were subsequently shown to protect against syngeneic and allogeneic tumors in other experimental models, including nude mice xenografted with human cervical carcinoma cells [7-9]. An international registry (IRCC) has been recently established with the aim to report results from current clinical trials using CIK cells, either as such or additionally manipulated [10]. Eleven clinical trials with autologous or allogeneic CIK cells were identified, with 426 patients enrolled. Most trials included male patients with hepatocellular carcinoma, gastric cancer and relapsed lymphoma [11,12]. A clinical response was reported in 384 patients who received up to 40 infusions of CIK cells. The total response rate was 24% and a decrease of tumour volume was documented in 3 patients. However, disease-free survival rates were significantly higher in patients treated with CIK cells than in a control group without CIK treatment.

Thymoglobulin^® ^(TG) is a purified, pasteurized preparation of polyclonal γ immunoglobulin raised in rabbits against human thymocytes [13]. TG is currently indicated for the prevention and/or treatment of renal transplant rejection, and displays specificity towards a wide variety of surface antigens on both immune system and endothelial cells. The precise mechanism(s) of action underlying its immunosuppressive efficacy are unclear, although T-cell depletion is considered to play a prominent role. Other mechanisms include lymphocyte surface antigen modulation, transcription factor activation, and interference with processes of immune system cells, such as cytokine production, chemotaxis, endocytosis, stimulation and proliferation (reviewed in ref. [13]). TG may also induce apoptosis, antibody-dependent lysis or complement-mediated lysis of various immune system cells, thus negating leukocyte-endothelial cell adhesion. Intriguingly, anti-lymphocyte globulin therapy in patients with aplastic anemia enhanced the function of MHC-unrestricted lymphocytes [14]. It is presently unknown whether TG can expand CIK cells more efficiently than αCD3 mAb in clinical-grade cultures.

We report herein the results of an *in vitro *study where TG was confronted with αCD3 mAb for its ability to promote the expansion and acquisition of cytotoxicity by CIK cells. We show that TG amplifies the number of CIK cells with greater efficiency than αCD3 after 21 days in culture. CIK cells generated in this fashion express a constellation of NK cell-associated inhibitory/activating receptors, release considerable amounts of IL-12p40 and lyse the NK-sensitive K562 cell line. The above culture conditions were also applied to PBMC from heavily pre-treated cancer patients, to ascertain whether TG can be a candidate drug for the optimization of CIK expansion protocols in preparation for clinical trials.

## Materials and methods

### Generation of CIK cells

CIK cells were generated under good manufacturing practice (GMP) conditions. Peripheral blood samples were obtained by phlebotomy in 10 consented healthy donors (median age 45 years; range, 22-58 years) and by steady-state apheresis in 4 patients with advanced cervical cancer (n = 3) or melanoma (n = 1). The patients' characteristics are listed in Table 1. The investigations were reviewed and approved by the Ethical Committee of the Catholic University Medical School in Rome (protocol ID: P/757/CE/2009).

**Table 1:** Patients' characteristics.

UPN	Age/Sex	Tumor (histotype)	Stage/grade at diagnosis	Previous treatments	WBC×10^3^/μl (PB/LK)*	Lymphocytes×10^3^/μl (PB/LK)*
1	30/F	Melanoma	Advanced, metastatic disease	Surgery, chemotherapy	4.8/55.1	1.19/28.82

2	62/F	Cervical cancer (squamous)	FIGO IIB	Neoadjuvant radiochemotherapy, radical surgery, chemotherapy (2 lines)	5.0/66.2	1.28/33.9

3	44/F	Cervical cancer (squamous)	FIGO IB	Radical surgery, adjuvant radiochemotherapy, chemotherapy (4 lines)	5.52/29.8	0.69/14.66

4	55/F	Cervical cancer (squamous)	FIGO IIIB	Radiochemotherapy, chemotherapy (3 lines)	5.41/51.6	1.52/22.14

WBC = white blood cells; PB = peripheral blood; LK = leukapheresis product.

*Blood cell counts were obtained at patient enrolment.

Peripheral blood samples collected by venipuncture were layered over Ficoll-Paque^® ^(GE Healthcare Life Sciences; Milan, Italy) and peripheral blood mononuclear cells (PBMC) were separated by centrifugation at 1,400 rpm for 30 minutes, as already detailed [15]. After washings with PBS, PBMC were grown in serum-free medium (X-VIVO 10; Bio-Whittaker Europe, Belgium) supplemented with 80 mg/L gentamycin (Schering Plough, Milan, Italy) and incubated at 37°C in a 5% CO_2 _atmosphere. Cells were seeded at 2.0 × 10^6 ^cells/ml in 25 cm^2 ^cell culture flasks (Corning, NY 14831, USA). On day 0, cells were activated with recombinant human IFN-γ (1,000 IU/ml; Imukin^®^, Boehringer Ingelheim, Ingelheim, Germany). The following day, cells were stimulated with either αCD3 mAb (UCHT1 clone; 50-500 ng/ml, BD Biosciences, San Diego, CA) or Thymoglobulin^® ^(50-500 ng/ml, Genzyme Corp., Cambridge, MA) and recombinant human IL-2 (rHuIL-2, 300 IU/ml; Proleukin^®^, Novartis Pharma, Milan, Italy). Cell suspensions were maintained in subculture with fresh medium supplemented with rHuIL-2 every 3 days for 3 weeks. For quality control, aliquots of cells were harvested weekly and used for automatic cell counting, phenotypic analysis, and microbiologic testing. Cell viability was evaluated at the end of the culture period by flow cytometry, after labeling with 7-amino-actinomycin-D (7-AAD; Sigma-Aldrich, Milan, Italy) [16].

### Flow cytometry and immunofluorescence

At baseline (day 0) and after 7, 14 and 21 days in culture, aliquots of cells were incubated for 30 minutes at 4°C with fluorochrome-conjugated mAb to CD3, CD8, CD45, CD16+CD56 (BD Multitest™IMK Kit; BD Biosciences, Mountain View, CA), CD94, CD158a (KIR2DL1), CD158b (KIR2DL2/DL3; BD Biosciences), NKG2A (KLRC1 or CD159a; R&D Systems, Oxon, UK), NKp46 (CD335), NKG2D (CD314; Beckman Coulter, Milan, Italy). Isotype-matched, fluorochrome-conjugated mAb from the same manufacturers were used to control for background fluorescence. The intracellular expression of the FoxP3 transcription factor was detected in fixed/permeabilized T cells that were initially labeled with anti-CD4 and anti-CD25 mAb (both from BD Biosciences), followed by Alexa Fluor 488-conjugated rat anti-human FoxP3 mAb (PCH101 clone; Human Regulatory T Cell Staining Kit; eBioscience, San Diego, CA). Cells were run through a FACS Canto^® ^flow cytometer (BD Biosciences) with standard equipment [17]. Samples were analyzed with the FACS Diva^® ^software package (BD Biosciences).

### Cytotoxicity assay

After 21 days in culture, aliquots of cells were used for cytotoxicity assays. Calcein acetoxymethyl ester (CAM) has been recently developed as an alternative to radioactive ^51^Cr release assay [18]. CAM is a lipid-soluble, non-polar compound that passively crosses the plasma membrane in living cells, where it is cleaved by intracellular esterases to reveal a very polar derivative of fluorescein (calcein) that remains trapped in the cytoplasm. CAM (Fluka, Sigma Aldrich) was dissolved in DMSO to a final concentration of 1 mM and stored in aliquots at -80°C. K562 target cells (1 × 10^6^), derived from a patient suffering from CML in blast crisis, were incubated in X-VIVO 10 medium in the presence of pre-titrated concentrations of CAM (0.1 μM) for 10 minutes at 37°C, shielded from light. The labeled cells were washed two times in ice-cold medium supplemented with 10% fetal bovine serum (FBS), were re-suspended in X-VIVO 10 and then plated in round bottom 96-well plates at 5-10 × 10^5 ^cells/well in triplicate. CIK cells were added at the effector-to-target (E:T) ratios detailed in the Figure legends, in a final volume of 200 μl, and were incubated for 4 hours. Cells were then washed with ice-cold PBS and re-suspended in 20 μg/ml 7-AAD for 20 minutes at room temperature, shielded from light, before flow cytometry analysis [19]. 7-AAD is a fluorescent DNA dye that selectively binds to GC regions of the DNA. The 7-AAD assay has been used to detect the loss of membrane integrity during apoptosis of murine thymocytes and human peripheral lymphocytes [20]. Percent specific cell death was calculated according to the following formula, as previously published [21]:

$$\frac{\%\text{ dead targets }-\;\%\text{spontaneous dead targets}}{\text{1}00\;-\;\%\text{ spontaneous dead targets}}\times 100$$

### Measurement of IL-12p40

After 21 days, supernatants from CIK cell cultures were collected and used to quantify IL-12p40 production by enzyme-linked immunosorbent assay (ELISA; R&D Systems, Oxon, UK), as reported [22]. The limit of detection was <15 pg/ml IL-12p40.

### Statistical analysis

Data distribution was preliminarily tested with kurtosis and symmetry. Data were presented as median and inter-quartile range. All comparisons were performed with the Mann-Whitney or the Wilcoxon signed-rank tests for paired or unpaired determinations, as appropriate. The criterion for statistical significance was defined as *p *< 0.05.

## Results

### Generation of CIK cells with TG

**Figure 1:**
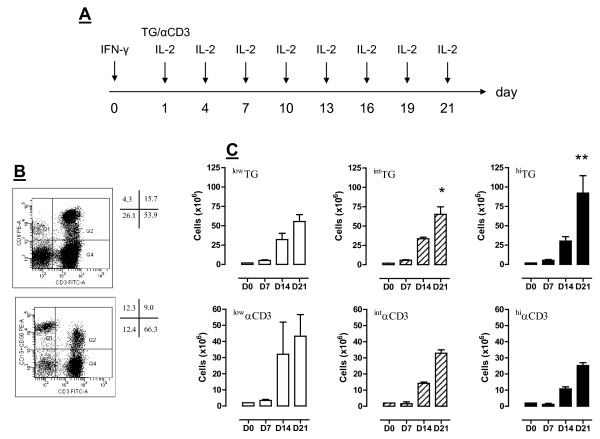
**Experimental layout and expansion of PBMC in cultures supplemented with TG**. *Panel A*: PBMC from consented healthy donors were initially exposed to IFN-γ (day 0), followed by different concentrations of either TG or αCD3 mAb (day +1) and IL-2 every 3 days. Further details are provided in Materials and Methods. *Panel B*: The frequency of CD3^+^CD8^+ ^T cells, NK cells (CD3^-^CD16^+^CD56^+^) and CD3^+^CD56^+ ^T cells from a representative PBMC sample at baseline is shown. Quadrant markers were set according to the proper isotypic control (not shown). The percentage of cells staining positively for a given antigen is indicated. *Panel C*: Cells were harvested weekly and counted. The number of cells was significantly higher after challenging with TG either at 250 (^int^TG; *p < 0.05) or 500 ng/ml (^hi^TG; **p < 0.05) compared with equal concentrations of αCD3 mAb (bottom row).

In a first set of experiments, we determined whether and to what extent TG promotes the generation of functional CIK cells and other desirable populations of immune effectors, namely, CD3^+^CD8^+ ^T cells and CD3^-^CD56^+ ^NK cells, starting from PBMC preparations. To this end, PBMC from consented volunteer donors were cultured in the presence of IFN-γ, IL-2 and either TG or αCD3 mAb at low (50 ng/ml), intermediate (250 ng/ml) or high concentration (500 ng/ml), as schematically depicted in Figure 1A. Cells were harvested on days +7, +14 and +21, were counted to calculate fold-expansion compared with baseline and were used to assess informative phenotypic features. The percentage of CD3^+^, CD8^+ ^and CD3^+^CD56^+ ^T cells in a representative PBMC sample before culturing is shown in Figure 1B. When used at intermediate (^int^TG) and high concentration (^hi^TG), TG induced a greater expansion of PBMC compared with equal concentrations of αCD3 mAb, and the difference was maximal after 14 and 21 days in culture (Table 2 and Figure 1c). ^Hi^TG promoted a 46.08-fold expansion of PBMC on day +21, compared with a median 11.75-fold expansion in the presence of ^hi^αCD3 mAb. In contrast, ^int^αCD3 and ^hi^αCD3 mAb failed to further increase PBMC number compared with ^low^αCD3 at any time-point in culture (Table 2), likely reflecting enhanced levels of activation-induced cell death. As shown in Table 2, both ^int^TG and ^hi^TG caused a greater fold-expansion of PBMC compared with αCD3 mAb at a concentration routinely used to differentiate CIK cells, i.e., 50 ng/ml.

**Table 2:** TG-induced expansion (fold-increase) of PBMC from healthy donors.

Culture condition	T = 7d	T = 14d	T = 21d
^low^αCD3 (50 ng/ml)	1.70 (1.2-2.3)	8.47 (3.9-15.58)	22.21 (9.78-33.04)

^low^TG (50 ng/ml)	2.90 (1.72-2.94)	8.74 (7.85-16.61)	30.56 (18.91-33.65)

			

^int^αCD3 (250 ng/ml)	0.30 (0.24-1.35)	2.63 (0.26-5.01)	14.3 (10.05-15.41)

^int^TG (250 ng/ml)	2.50 (2.47-3.56)	14.86*^,^^ (7.21-17.45)	33.47^§,^^ (23.72-40.77)

			

^hi^αCD3 (500 ng/ml)	0.59 (0.28-0.9)	5.28 (5.03-8.30)	11.75 (9.80-12.05)

^hi^TG (500 ng/ml)	2.63 (2.05-3.02)	11.96**,^ (6.01-17.91)	46.08^§§,^^^ (34.84-57.31)

Fold expansion of PBMC cells in culture has been calculated by dividing the absolute number of cells at days 7, 14 and 21 by the absolute number of cells at day 0. * and ^§^p < 0.05 compared with ^int^αCD3 mAb; ** and ^§§ ^p < 0.01 compared with ^hi^αCD3 mAb. ^ p < 0.05 compared with ^low^αCD3 mAb; ^^ p < 0.01 compared with ^low^αCD3 mAb.

**Figure 2:**
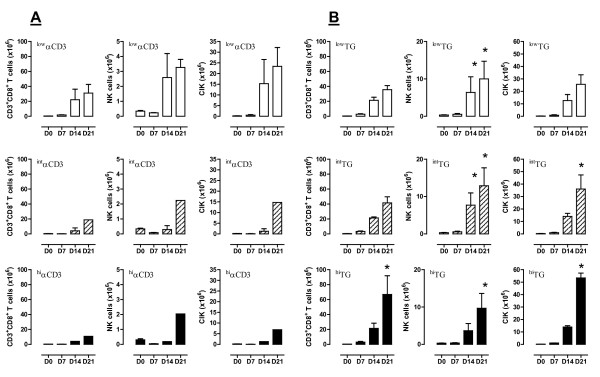
**Expansion of CIK cells, NK cells and CD8^+ ^T cells in cultures supplemented with TG**. The absolute number of CD3^+^CD8^+ ^T cells, NK cells (CD3^-^CD16^+^CD56^+^) and CIK cells (CD3^+^CD16^+^CD56^+^) was estimated weekly after the provision of either αCD3 mAb (panel A) or TG (panel B) to the cultures. Cumulative results from 10 experiments performed with 10 different PBMC preparations are expressed as median and inter-quartile range. *denotes a statistically significant difference (p < 0.05) when comparing cell numbers in TG-containing cultures with those in cultures nurtured with an equal concentration of αCD3 mAb.

**Figure 3:**
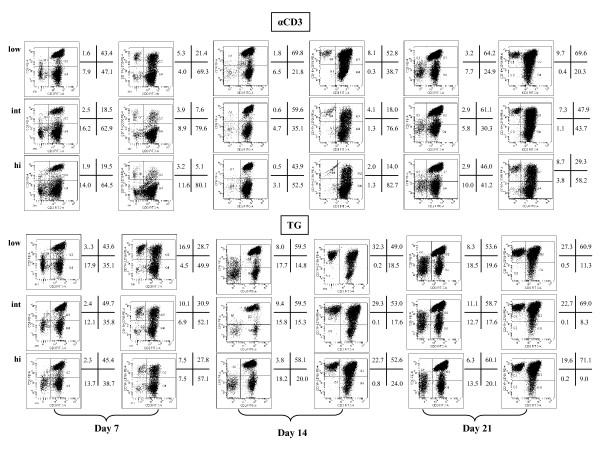
**Phenotypic features of TG-expanded CIK cells, NK cells and CD8^+ ^T cells**. The frequency of CD3^+^CD8^+ ^T cells, NK cells (CD3^-^CD16^+^CD56^+^) and CIK cells (CD3^+^CD16^+^CD56^+^) was measured by flow cytometry weekly after the provision of different concentrations of either TG or αCD3 mAb to the cultures. One experiment out of 10 with similar results is shown. Quadrant markers were set according to the proper isotypic control (not shown). The percentage of cells staining positively for a given antigen is indicated.

We next calculated the absolute number and estimated the frequency of CD3^+^CD8^+ ^T cells, CD3^-^CD16^+^CD56^+ ^(NK cells), and CD3^+^CD16^+^CD56^+ ^(CIK cells) in cultures supplemented with αCD3 mAb (Figure 2A; Figure 3) or TG (Figure 2B; Figure 3). These PBMC cultures started with a typical percentage of approximately 6-9% and 8-12% CD3^+^CD56^+ ^T cells and NK cells, respectively (Figure 1B). After the 21-day culture period, the median percentages of CIK cells and NK cells in cultures maintained with ^hi^αCD3 and ^hi^TG were 64% and 9.7%, and 55% and 27.5%, respectively. As expected, CIK cells were predominantly comprised of CD3^+^CD8^+ ^T cells. It should be noted that the percentage of CD3^+^CD8^+ ^T cells at any time-point was consistently higher in cultures supplemented with TG. This difference was maximal when comparing CIK cultures at day +7 after priming with TG or αCD3 mAb, as illustrated in Figure 2A-2B (cumulative data) and in Figure 3 (a representative experiment out of 10 with similar results). At this time-point in culture, the increase of αCD3 mAb concentration in the medium was associated with a progressive decline in the percentage of CD3^+^CD8^+ ^T cells, a phenomenon that was also evident after 14 and 21 days (Figure 3). Similarly, NK cells were significantly more represented within CIK cultures activated with TG when compared with cultures nurtured with αCD3 mAb. Whereas day-21 CIK cultures contained a median 27.5% NK cells after priming with ^hi^TG, the fraction of NK cells was consistently < 10% in CIK cultures activated with αCD3, irrespective of the mAb concentration in the culture medium (Figure 3). Taken together, phenotypic analyses indicated that the heterogeneous population of cells that emerged after 21 days in culture with TG contained higher numbers of CIK cells and other immune effectors such as CD8^+ ^T cells and NK cells compared with those differentiated with αCD3 mAb. Also, ^hi^TG was significantly more effective than ^int^TG and ^low^TG at generating the three populations of immune effector cells.

**Figure 4:**
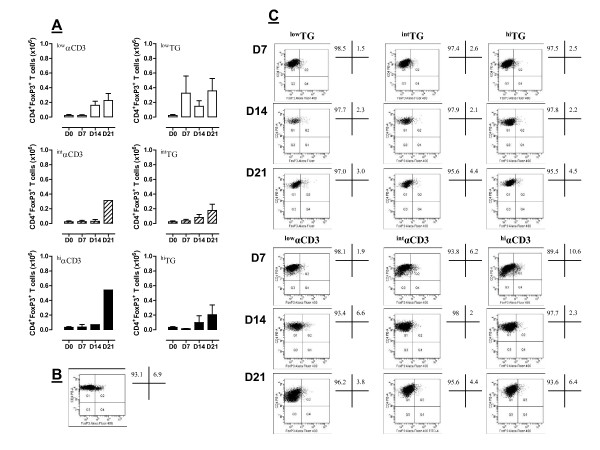
**Frequency of *bona fide *Treg cells after the provision of either αCD3 mAb or TG to the cultures**. *Panel A*: Cumulative frequency of CD4^+^FoxP3^+ ^Treg cells within PBMC stimulated with either TG or αCD3 mAb. Data are expressed as median and inter-quartile range. *Panels B and C*: Flow cytometry detection of intracellular FoxP3 in CD4^+ ^T cells at baseline (B) and after their *in vitro *expansion (C). Cells were fixed, permeabilized and labeled as detailed in Materials and Methods. Quadrant markers were set according to the proper isotypic control (not shown). The percentage of cells staining positively for intracellular FoxP3 is indicated.

We next addressed whether TG in combination with IL-2 favors the concomitant expansion of Treg cells, as defined by their FoxP3^+ ^phenotype. The rationale for these experiments stems from a previous report indicating that high concentrations of TG (10 μg/ml) up-regulate molecules associated with Treg function on CD4^+ ^T cells [23]. Even more intriguingly, IL-2, which is routinely used to generate CIK cells, is a Treg-cell growth factor both *in vitro *(reviewed in ref. [24]) and *in vivo *[25,26]. As shown in Figure 4A, the cumulative frequency of *bona fide *Treg cells was lower in cultures containing TG versus αCD3, suggesting that the clinical application of TG for the generation of anti-tumor effector cells is not expected to negatively affect anti-tumor immunity through Treg cells. A representative experiment aimed at quantifying Treg-cell frequency by flow cytometry both at baseline and in expanded CIK cultures is illustrated in Figure 4B and 4C. Based on the above data and to maximize the yield of CIK cells in culture, TG was consistently used at 250 ng/ml or 500 ng/ml in all subsequent experiments, as detailed in the Figure legends.

### Phenotype and effector functions of *in vitro*-generated CIK cells

**Figure 5:**
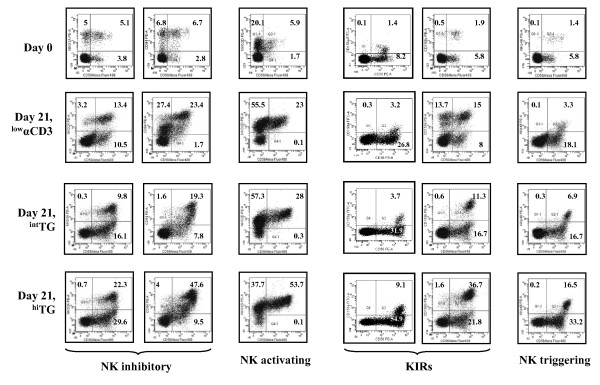
**Expression of NK-cell inhibitory/activating receptors on CIK cells generated with TG**. After 21 days of culture in the presence of either TG or αCD3 mAb, cells were harvested and labeled with mAb recognizing NK inhibitory receptors (NKG2A/CD94), NK activating receptors (NKG2D), KIR (CD158a, CD158b) and NK triggering receptors (NKp46). A representative experiment out of 10 with similar results is shown. Quadrant markers were set according to the proper isotypic control (not shown). The percentage of cells staining positively for a given antigen is indicated.

We proceeded to investigate the expression of triggering and inhibitory receptors that may modulate cytotoxicity by the cultured CIK cells. To this end, PBMC were primed with ^int^TG or ^hi^TG and then maintained for 21 days with IL-2 to achieve maximal expansion, followed by labeling with a panel of mAb recognizing the NK activating receptor NKG2D, the NK triggering receptor NKp46, the NK inhibitory receptor CD94-NKG2A and two representative members of the KIR family (KIR2DL1 or CD158a and KIR2DL2/DL3 or CD158b). The phenotypic features of CIK cells generated with TG were compared with those of CIK cells emerging from PBMC cultures containing ^low^αCD3, a standard culture protocol for CIK cells [27]. Cells were initially gated based on their expression of CD3. Data shown in Figure 5 are representative of the co-expression of CD56 and the antigens of interest on CD3^+ ^T cells harvested from the PBMC cultures at day +21. ^Hi^TG induced significantly higher levels of KIR on the expanded CIK cells, when compared with either ^int^TG or ^low^αCD3 mAb (Figure 5A). Similarly, the NKG2A/CD94 heterodimer, the NKp46 triggering receptor and the NKG2D activating receptor were preferentially up-regulated on CIK cells differentiated with ^hi^TG compared with the other culture conditions herein established (Figure 5).

**Figure 6:**
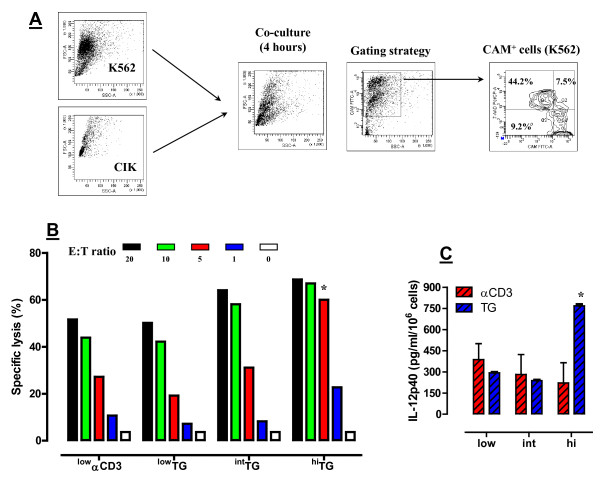
**Cytolytic function and IL-12p40 release by CIK cells generated with TG**. *Panel A: *The gating strategy for the analysis of CIK-mediated cytotoxicity is shown in a representative experiment. After co-culture with CIK cells, K562 targets were identified and gated based on CAM expression. The percentage of lysed K562 cells was then calculated on a CAM/7-AAD contour plot. *Panel B*: After 21 days of culture in the presence of either ^low^αCD3 mAb or different concentrations of TG, cells were harvested and co-cultured with NK-sensitive tumor cell targets (K562 cells) for 4 hours at the indicated effector-to-target (E:T) ratio. K562 cells were pre-labeled with CAM, a fluorescent probe. The percent specific lysis was calculated as detailed in Materials and Methods. * denotes a *p *value < 0.01 when compared with cultures containing ^int^TG, ^low^TG and ^low^αCD3 mAb. Panel C: IL-12p40 release was measured at the end of culture (21 days) in the presence of escalating concentrations of either αCD3 mAb or TG. Bars depict median values recorded in 3 independent ELISA run in duplicate with supernatants from 3 different PBMC preparations. * denotes a *p *value < 0.01 when compared with cultures containing ^int/low^TG, ^int/low^αCD3 mAb and ^hi^αCD3.

A further set of experiments was devoted to the analysis of CIK cell cytotoxicity against the NK-sensitive K562 target cells, taking advantage of a non-radioactive, flow cytometry-based assay. K562 cells were loaded with the fluorescent probe CAM and then co-cultured with escalating numbers of CIK cells, as detailed in Materials and Methods. Cells emerging from the co-cultures were gated based on CAM fluorescence and then visualized on a CAM/7-AAD contour plot to enumerate CAM^+^7-AAD^+ ^dead targets (Figure 6A). In accordance with phenotypic data showing a higher expression of NK effector molecules on cells harvested from TG-driven cultures, CIK cells differentiated with ^int^TG and ^hi^TG lysed K562 cells more efficiently than CIK cells generated with ^low^αCD3 mAb (Figure 6B). The cytotoxicity of CIK cells cultured under ^hi^TG was maximal at an E:T ratio of 10. It should be noted that the difference in cytotoxic potential of CIK cells expanded by ^hi^TG was most pronounced at an E:T ratio of 5, where specific lysis averaged 60% compared with <30% under the other culture conditions (p < 0.01; Figure 6B). These observations suggest that a higher frequency of cytotoxic cells was present within the population of PBMC expanded with ^hi^TG compared with either ^int/low^TG or ^low^αCD3 mAb.

IL-12 is a T helper type 1 (Th1) cytokine that augments NK-cell proliferation *in vitro *and enhances their cytotoxicity *in vivo *[28]. The expression of IL-12p40 subunit is known to be restricted to cells that produce the biologically active IL-12 heterodimer [29]. As shown in Figure 6C, IL-12p40 levels were significantly higher in day 21-cultures differentiated with ^hi^TG compared with either lower doses of TG or αCD3 mAb. Taken together, these experiments suggest that ^hi^TG-differentiated CIK cells may be particularly suitable for adoptive immunotherapy approaches to cancer.

### Generation and function of CIK cells from cancer patients

**Figure 7:**
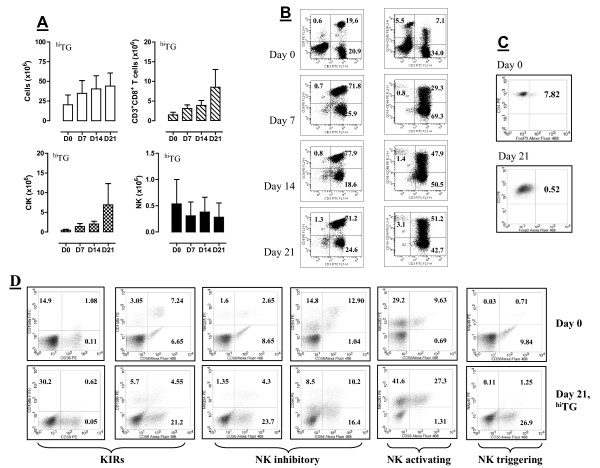
**Generation of CIK cells with ^hi^TG from patients with advanced solid cancer**. The culture conditions described in Materials and Methods were used to generate CIK cells from the PBMC of 4 patients with advanced cancer. ^Hi^TG was used in these studies because it induced maximal expansion of CIK cells from healthy donor PBMC. *Panel A*: The absolute number of PBMC, CD3^+^CD8^+ ^T cells, NK cells (CD3^-^CD16^+^CD56^+^) and CIK cells (CD3^+^CD16^+^CD56^+^) was estimated weekly after the provision of ^hi^TG to the cultures. Results summarize (median and inter-quartile range) 4 independent experiments performed with PBMC preparations from 4 different patients. *Panel B*: The frequency of CD3^+^CD8^+ ^T cells, NK cells (CD3^-^CD16^+^CD56^+^) and CIK cells (CD3^+^CD16^+^CD56^+^) from a representative PBMC sample is shown at baseline (day 0) and after 7, 14 and 21 days in culture. Quadrant markers were set according to the proper isotypic control (not shown). The percentage of cells staining positively for a given antigen is indicated. *Panel C*: Flow cytometry detection of intracellular FoxP3 in CD4^+ ^T cells from a representative PBMC culture. Cells were fixed, permeabilized and labeled as detailed in Materials and Methods. The percentage of cells staining positively for intracellular FoxP3 is indicated both at baseline and after 21 days in culture. Quadrant markers were set according to the proper isotypic control (not shown). *Panel D*: The expression of NK-cell inhibitory/activating receptors was investigated by flow cytometry, as previously detailed. A representative experiment out of 4 with similar results is shown. Quadrant markers were set according to the proper isotypic control (not shown). The percentage of cells staining positively for a given antigen is indicated.

**Figure 8:**
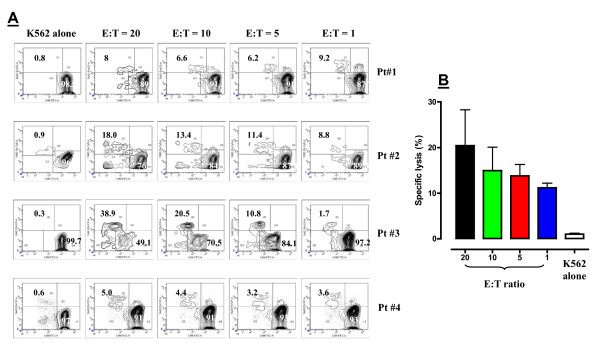
**Cytolytic activity of CIK cells generated with ^hi^TG from patients with advanced solid cancer**. *Panel A*: CIK cells were differentiated with ^hi^TG from 4 patients with advanced solid cancer and were used to assess cytolytic activity against NK-sensitive tumor-targets. K562 CML cells were pre-loaded with CAM, a fluorescent probe, followed by their co-culture with CIK cells for 4 hours at the indicated E:T ratio. Contour plots depict the raw percentage of 7-AAD^+^CAM^int ^target cells that have been lysed at the end of the 4-hour co-culture. Quadrant markers were set according to the proper isotypic control (not shown), i.e., K562 cells that were neither loaded with CAM nor labeled with 7-AAD. *Panel B*: Cumulative cytotoxicity of CIK cells differentiated from the 4 patients with advanced solid cancer. Bars depict median values with interquartile range. The percentage of 7-AAD^+ ^cells in cultures with K562 target cells alone (background cell death) is shown as uncolored column.

In view of the promising results obtained when challenging PBMC from healthy donors with ^hi^TG, we evaluated whether the generation of CIK cells from cancer patient-derived PBMC could be successfully pursued under the same experimental conditions (priming with IFN-γ on day 0 and then with IL-2 and TG on day +1). Figure 7A depicts the average number of PBMC, CD8^+ ^T cells, NK cells and CIK cells in 4 experiments performed with PBMC from 4 patients with cervical cancer or melanoma. ^Hi^TG induced a vigorous expansion of PBMC, CD8^+ ^T cells and CIK cells, but not NK cells, peaking after 21 days in culture (Figure 7A). It should be pointed out that the average number of NK cells differentiated from patient PBMC was lower compared with donor PBMC at any time-point. Nevertheless, these data suggest that TG can generate clinically relevant numbers of CIK cells in cancer-bearing patients. Table 3 summarizes the frequency of all types of effector cells that were differentiated from patients' PBMC after 21 days in culture. The frequency of CD8^+ ^T cells, NK cells and CIK cells at baseline and after 7, 14 and 21 days in culture in a representative experiment is shown Figure 7B. As depicted in Figure 7C and in line with our findings with PBMC from healthy volunteers, the percentage of *bona fide *Treg cells was significantly lower after culturing with any concentration of TG for 21 days compared with the frequency measured in patients' peripheral blood, indicating *in vitro *depletion of pre-existing Treg cells. The higher percentage of Treg cells routinely detected in baseline peripheral blood samples was not unexpected, based on previously published data on the expansion of the Treg compartment in cancer patients [30]. Importantly, patient-derived CIK cells expressed lower levels of KIR, NKG2A, NKG2D and NKp46 compared with CIK cells differentiated from normal donors (Figure 7D). Functional assays are individually shown in Figure 8A and indicated that *in vitro *K562 cell lysis by CIK cells was highly efficient in 2 out of 4 cases here examined (patients #2 and #3), especially when CIK cells were plated at a relatively high E:T ratio. The cytotoxicity experiments performed with CIK cells from the 4 patients enrolled in the present study have been summarized in Figure 8B. Both patients whose CIK cells were capable of lysing K562 cells *in vitro *were affected by cervical carcinoma, but had been heavily pre-treated and had advanced, metastatic disease at study enrolment (Table 1). No obvious differences in terms of white blood cell and lymphocyte count at baseline (day 0, i.e., at time of leukapheresis) were evident when comparing patients #2 and #3 with the 2 patients (#1 and #4) showing poor *in vitro *cytolytic responses (Table 1), suggesting that qualitative rather than quantitative determinants likely accounted for the observed phenomena. It should be noted that CIK cultures from patient #3 were particularly heterogeneous and contained a relatively high percentage of *bona fide *NK cells with a classical CD3^-^CD56^+ ^phenotype.

**Table 3:** Phenotypic features of patient-derived effector cells after 21 days in culture.

Pt #	CD3^+^CD8^+ ^(T cells)	CD3^+^CD16^+^CD56^+ ^(CIK cells)	CD3^-^CD16^+^CD56^+^ (NK cells)
1	78%	57.64%	0.5%

2	81.5%	40.5%	1.2%

3	79.8%	36.3%	12.2%

4	71.2%	51.2%	3.1%

## Discussion

The present study aimed at dissecting the role of TG in the differentiation of CIK cells, a heterogeneous population of immune effector cells sharing T-cell and NK-cell characteristics. The relationship between *in vivo *circulating CD3^+^CD56^+ ^T cells and *in vitro*-generated CIK cells is poorly understood. Human CD3^+^CD56^+ ^T cells can be detected within peripheral blood CD8^+ ^T cells and express CD16, CD161, NKG2D and KIR such as CD158a, CD158b and CD94 [31]. The most extensively characterized human NK antigen-expressing CD3^+ ^T-cell subset is represented by CD56^+ ^T cells that account for ~5% of peripheral blood T cells. CD56^+ ^T cells lyse NK-sensitive target cell lines *in vitro*, can be selectively expanded by IL-2 and IL-15, but require cell activation to trigger the secretion of effector cytokines such as IFN-γ and TNF-α. It has been recently shown that CIK cells expanded with IFN-γ, OKT3 and IL-2 resemble activated effector-memory CD8^+ ^T cells and likely derive from CD56^- ^T cells, as suggested by gene expression profiling [32]. In this respect, only 50 differentially expressed genes were identified when comparing CIK cells and CD56^- ^T cells, whereas 115 genes were either up-regulated or down-regulated in CIK cells compared with CD56^- ^T cells [32]. Collectively, it is now recognized that CIK cells have undisputed advantages over other cell therapy products that make them particularly attractive, such as ease of *in vitro *expansion, superior *in vivo *activity than LAK cells, and no need for exogenous administration of IL-2 for *in vivo *priming [33,34]. Current laboratory protocols dictate that CIK cells should be differentiated with IFN-γ and the OKT3 mAb to CD3, followed by repeated additions of IL-2 for a maximum of 21-28 days [3,11,12,33].

Our interest in TG as a candidate drug to expand CIK cells in preparation for clinical trials originated from reports indicating that binding of TG to CD16, CD18 and NKp46 on NK cells potentiates their activation and degranulation, and enhances IFN-γ production, although this translated into the decrease of NK cytotoxicity against K562 cells [35]. When selecting the optimal TG concentration to be used in culture, we took advantage of previously published papers showing the following points. First, TG may induce ~ 15% NK cell apoptosis *in vitro*, when added at concentrations ranging from 1 μg/ml to 100 μg/ml [35,36]. Second, TG directly affects CD4^+ ^T-cell function and cytokine release when used at 10 μg/ml, transiently up-regulating CD25, FoxP3 and CTLA-4 mRNA and protein, and increasing IL-2, IL-4, IL-10 and IFN-γ secretion in culture supernatants. Third, CD4^+ ^T cells pre-treated with 10 μg/ml TG inhibit the proliferation of autologous CD4^+ ^T cells to allogeneic PBMC, suggesting the acquisition of a regulatory phenotype [23]. We therefore elected to provide TG at relatively low concentrations (from 50 to 500 ng/ml) to the PBMC cultures, in order to minimize both NK and possibly CIK-cell apoptosis as well as the amplification of Treg cell numbers. TG significantly expanded PBMC compared with ^low^αCD3 mAb, leading to the *in vitro *generation of a heterogeneous population comprised of CD8^+ ^T cells, NK cells and CIK cells. Especially when used at 500 ng/ml, TG augmented the proliferation of PBMC with subsequent enhanced generation of CD8^+ ^T cells, NK and CIK cells, compared both with an equally high concentration of αCD3 mAb and with ^low^TG or ^int^TG. This implies that ^hi^TG may be particularly effective at the concurrent expansion of all three types of immune effector cells, namely, CD8^+ ^cytotoxic T cells, NK cells and CIK cells, at variance with αCD3 mAb. Of potential importance for the design of clinical trials with TG/IL-2-expanded CIK cells, the frequency of *bona fide *Treg cells at any time-point in culture was similar when comparing PBMC preparations activated with IL-2 and TG or αCD3 mAb, thus reassuring against the infusion of excessive numbers of tumor-suppressive Treg cells [25].

NK cells express a wide array of inhibitory and activating receptors such as KIR, NKG2A/CD94, NKG2D, NKp46 and others, which recognize both foreign and self antigens expressed by target cells, and finely regulate NK cytotoxicity against virus-infected and tumor cells [37]. NK receptors play a crucial role in innate immunity against infections and in anti-tumor immune responses. It is presently unknown whether TG modulates the expression of NK receptors on CIK cells, a finding with important implications for their cytotoxic activity and for their ability to combat infections. The KIR family consists of 11 highly polymorphic receptors that are clonally distributed on NK cells and bind directly to classical MHC molecules such as particular HLA-Cw alleles. KIR may be expressed at low levels (i.e., < 10%) on CIK cells differentiated with standard protocols [32]. In our study, both CD158a (KIR2DL1) and CD158b (KIR2DL2/DL3) were readily detected on CIK cells expanded with ^hi^TG, with expression levels ranging from ~15% to ~65% of CD3^+^CD56^+ ^cells for CD158a and CD158b, respectively. Although KIR-expressing CD8^+ ^T cells exist in human peripheral blood [38], the stimuli that regulate KIR induction in T cells are poorly defined [39], and may include demethylation events [40]. Interestingly, engagement of CD158b by MHC ligands on human CD8^+ ^effector T cells hinders TCR signaling and limits T-cell proliferation [41]. Based on our findings, it is tempting to speculate that TG provided an *in vitro *signal orchestrating the expression of KIR on CIK cells. Conceivably, the TG-driven expression of KIR might represent a feedback signal to limit excessive CIK expansion and/or uncontrolled *in vitro *cell death. Although the nature of the signal(s) delivered to CIK cells through TG remains to be identified, it is unlikely that cytokine stimuli such as IL-15 are implicated, based on our observation that IL-15 provision to CIK cultures did not translate into any further induction of KIR (Rutella S, unpublished observations, 2010). Our statement is also supported by a previous report demonstrating the inability of IL-15 and IL-21 to induce KIR expression on cord blood-derived NK progenitor cells [42].

*NKG2D *encodes for a lectin-related protein expressed as a homodimer and functioning as an activating receptor for ligands often expressed by tumor cells, namely, class I MHC-related molecules such as MICA, MICB, and UL16-binding proteins [43]. The NKG2A/CD94 receptor contains C-type lectin ectodomains, binds to HLA-E, a non-classical MHC protein important for viral surveillance, and functions as an inhibitory receptor by signaling through ITIM motifs [44,45]. As recently proposed, high surface levels of NKG2A/CD94 may be required to avoid excessive NK cell-mediated killing of HLA-E-bearing normal target cells [45]. Of interest, CD94/NKG2A expression on CD8^+ ^T cells may protect from apoptosis and favor the eventual emergence of memory T-cell responses [46]. In light of these findings, it is conceivable that high levels of CD94/NKG2A and KIR on TG-differentiated CIK cells promote cell survival, leading to protection from CIK-mediated killing of normal cells.

NKp46 belongs to a family of activating natural cytotoxicity receptors (NCR) for tumor cells [47], also including NKp30 and NKp44, that enables a precise identification of all NK cells. Upon engagement by specific ligands, NCR induce a strong activation of NK-mediated cytotoxicity, thus playing a pivotal role in tumor cell killing [48]. To date, NCR have been detected on NK cells in a restricted fashion and regardless of NK-cell activation status. Notably, NKp46 was found on ~15-20% of CD3^+ ^CIK cells differentiated with ^hi^TG, and lower expression levels of NKp46 correlated with lower TG concentrations in the culture medium. These data are backed by a recent study documenting a 10-20% expression of NKp30, NKp44 and NKp46 on CIK cells driven by IFN-γ, OKT3 and IL-2 [32]. Overall, these observations question the specificity of NCR for cells of the NK lineage and suggest that NCR may also contribute to the killing activity of CIK cells. When evaluated for their ability to lyse tumor targets, CIK effectors differentiated with TG were significantly more effective at killing K562 cells compared with those nurtured with αCD3 mAb. It should be noted that patient-derived CIK cells expressed lower levels of activating/inhibitory NK receptors and manifested a reduced lytic activity *in vitro *in 2 out of 4 cases. Although the very small number of patients under study precludes any sensible conclusion, it is likely that the generation of fully functional CIK cells by TG was hindered by immune suppressive circuits in patients with advanced metastatic disease.

IL-12, a prototype member of a family of IL-12-related cytokines that includes IL-23 and IL-27, is an instigator of Th1 immune responses and possesses *in vivo *anti-tumor activities [49]. IL-12 is a heterodimer formed by a 35-kDa light chain (known as p35 or IL-12α) and a 40-kDa heavy chain (known as p40 or IL-12β). Messenger RNA encoding IL-12p35 is present in many cell types, whereas mRNA encoding IL-12p40 is restricted to cells that produce the biologically active heterodimer [29]. Importantly, CIK cells generated with ^hi^TG released higher quantities of IL-12p40 compared with the other culture conditions here established. This finding portends favorable implications for the use of ^hi^TG in the generation of CIK cells, given the established role of IL-12 in the promotion of anti-tumor immunity [49].

In conclusion, we propose that TG is an attractive drug to maximize the yield and anti-tumor potency of CIK cell preparations. The expansion of immune effector cells in response to a combination of IFN-γ, TG and IL-2 occurred in the absence of a significant induction of Treg cells, whose infusion into cancer-bearing patients would be highly undesirable. From a clinical standpoint, CIK cells are likely to be efficacious at disease stages where the tumor burden is relatively low or in an adjuvant setting, rather than in advanced disease [10]. It is presently unknown whether the overall survival rate is significantly affected by this type of adoptive cellular therapy. Future studies will have to address whether CIK cells differentiated with TG offer advantages over those obtained with αCD3-based protocols and whether they may be integrated into current cancer treatments.

## Competing interests

The authors declare that they have no competing interests.

## Authors' contributions

GB made substantial contributions to conception and design and carried out the experiments; PI, AM, AP, AP and DF carried out the experiments; MC helped with some of the flow cytometry experiments; AP and GS contributed to study design and cared for the patients; LP contributed to study conception and design; SR made substantial contributions to conception and design, performed the experiments and the statistical analysis, analyzed and interpreted data and wrote the paper. All authors read and approved the final manuscript.
